# Supplementary material for: Analysis of chi angle distributions in free amino acids via multiplet fitting of proton scalar couplings
Source: Magn Reson (Gott). 2024 Aug 19;5(2):103–20. doi: 10.5194/mr-5-103-2024 (PMC11570886; doi:10.5194/mr-5-103-2024)
Supplement: The supplement related to this article is available online at: https://doi.org/10.5194/mr-5-103-2024-supplement. [file mr-5-103-2024-supplement.zip › Syed2024/data/fit1d_gissmo.tar.gz!/fit1d/arg/500/bmse000029_simulation_1_nmredata.zip!/bmse000029_simulation_1_nmredata/1H/pdata/1/index.html_C=M_O=A]

Index of /ftp/pub/bmrb/metabolomics/standards/L\_arginine/nmr/bmse000029/1H/pdata/1


# Index of /ftp/pub/bmrb/metabolomics/standards/L\_arginine/nmr/bmse000029/1H/pdata/1

|  | Name | Last modified | Size | Description |
| --- | --- | --- | --- | --- |
| --- | | | | |
|  | Parent Directory |  | - |  |
|  | hwcal.out | 02-Nov-2010 13:38 | 12 |  |
|  | outd | 02-Nov-2010 13:38 | 498 |  |
|  | 1i | 02-Nov-2010 13:39 | 128K |  |
|  | 1r | 02-Nov-2010 13:39 | 128K |  |
|  | auditp.txt | 02-Nov-2010 13:39 | 1.7K |  |
|  | intrng | 02-Nov-2010 13:39 | 86 |  |
|  | peaklist.xml | 02-Nov-2010 13:39 | 437 |  |
|  | peaks | 02-Nov-2010 13:39 | 672 |  |
|  | proc | 02-Nov-2010 13:39 | 1.8K |  |
|  | procs | 02-Nov-2010 13:39 | 1.7K |  |
|  | title | 02-Nov-2010 13:39 | 59 |  |
| --- | | | | |

Apache/2.2.3 (CentOS) Server at www.bmrb.wisc.edu Port 80
